# Supplementary material for: Onset of Bloch oscillations in the almost-strong-field regime
Source: Nat Commun. 2022 Dec 13;13:7716. doi: 10.1038/s41467-022-35401-3 (PMC9747800; doi:10.1038/s41467-022-35401-3)
Supplement: Supplementary file 1 — Supplementary Information [file 41467_2022_35401_MOESM1_ESM.pdf]

Supplementary Information

Onset of Bloch oscillations in the almost-strong-field regime

Jan Reislöhner[1], Doyeong Kim[1], Ihar Babushkin[2,3,4] and Adrian N. Pfeiffer[1]

[1]Institute of Optics and Quantum Electronics, Abbe Center of Photonics, Friedrich Schiller University, Max-Wien-Platz 1, 07743 Jena, Germany.
[2]Institute for Quantum Optics, Leibniz Universität Hannover, Welfengarten 1, 30167 Hannover, Germany.
[3]Cluster of Excellence PhoenixD (Photonics, Optics, and Engineering – Innovation Across Disciplines), Welfengarten 1, 30167 Hannover, Germany.
[4]Max Born Institute, Max Born Str. 2a, 12489 Berlin, Germany.

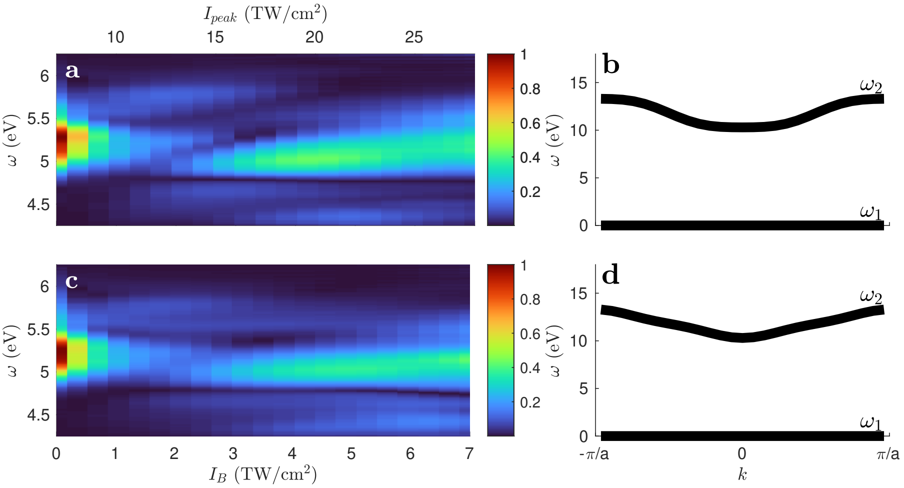

Supplementary Figure 1. The influence of the band shape. **a** and **c** are the same as Fig. 4**e** (Main Text), except that the shape of the conduction band Eq. (5, Main Text) has been replaced by $\omega_2^k = \frac{1}{2}b_1(1 - \cos(ka) - b_3(\cos(3ka) - \cos(ka)))$ with $b_3 = -0.1$ (**a**) and $b_3 = 0.1$ (**c**). The band shape is displayed in **b** for $b_3 = -0.1$ and in **d** for $b_3 = 0.1$.

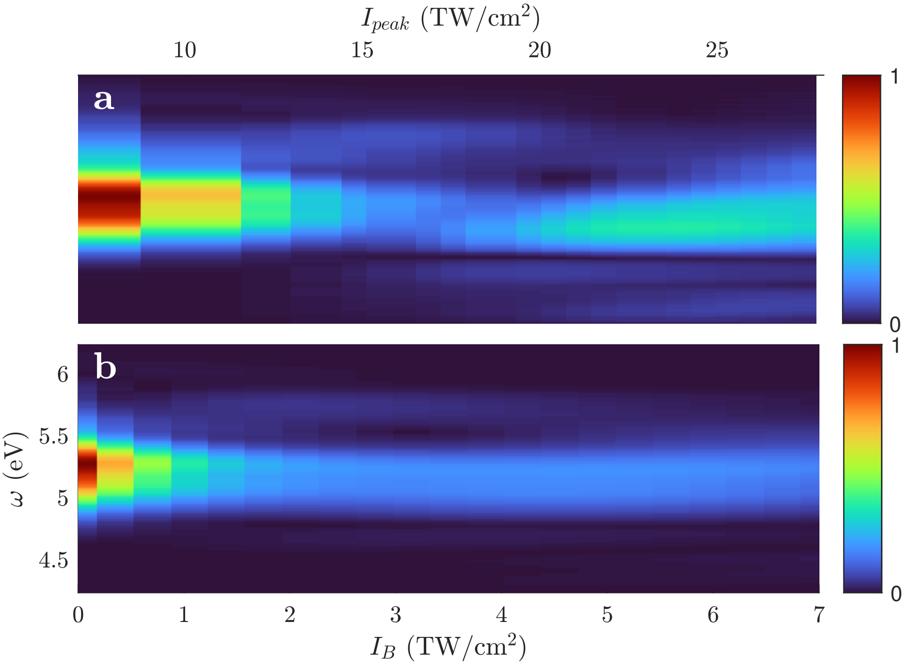

Supplementary Figure 2. Comparison of dephasing times. The same as Fig. 4**e** (Main Text), but using $T_2 = 10\,\text{fs}$ (**a**) and $T_2 = 3\,\text{fs}$ (**b**).

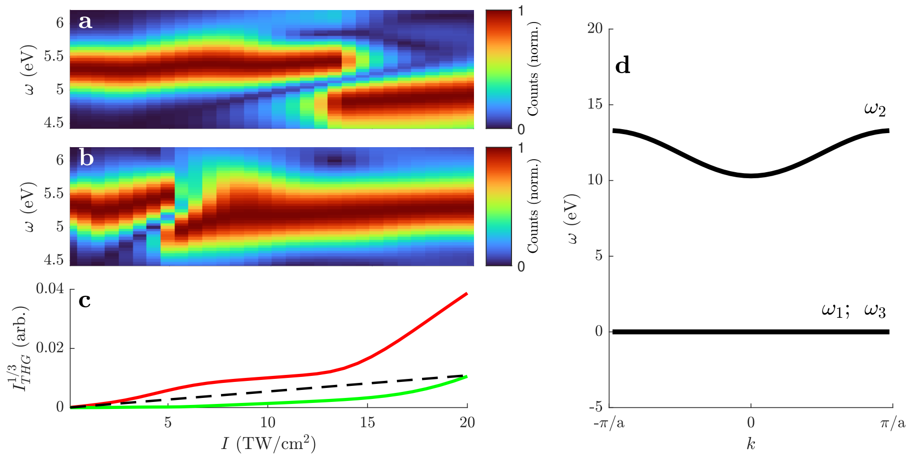

Supplementary Figure 3. The influence of numerical approximations. Time-domain integrations of Eq. (5, Main Text) using 8-fs Gaussian pulses in a three band model are performed. Unlike in the main text (Fig. 5), the same approximations as for the iterative series ($\rho_{11}^k - \rho_{22}^k = 1$; $\rho_{11}^k - \rho_{33}^k = 0$; $\rho_{33}^k - \rho_{22}^k = 1$) are used. The spectra of $P^{(\mathrm{NL})}$ and J are shown in **a** and **b**. The spectra have been normalized at each intensity to increase the visibility. The third root of the sum of $P^{(\mathrm{NL})}$ (red solid) and $J/3\omega_{12}$ (green solid) before normalization are depicted in (**c**), corresponding to the contribution of the nonlinear polarization and the current to the total THG intensity. For comparison, the black dashed line shows the instantaneous response $P^{(\mathrm{NL})} = \chi^{(3)}E^3$, which is a straight line in this diagram. The spectra and curves are almost identical to those in (Main Text) Fig. 5, justifying the approximations of the iterative series. The band structure used is shown in **d**.

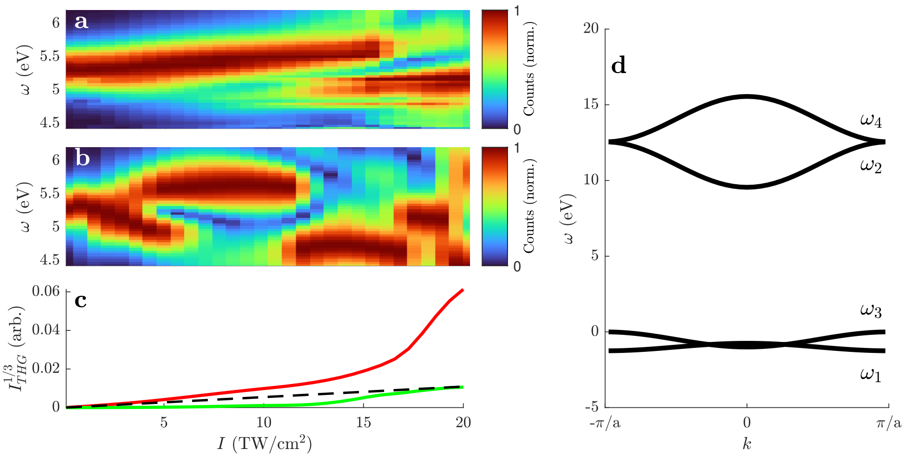

Supplementary Figure 4. Comparison to a more realistic band structure. Panels **a**, **b**, **c** are the same as in Supplementary Fig. 3 but using a more realistic band structure. Four bands are used as shown in **d**. The band energies are $\omega_n^k = \omega_n + \frac{1}{2}b_n(1-\cos(ka))$ with $\omega_n = -1$ eV; 9.3 eV; -1.25 eV; 15.3 eV and $b_n = -0.5$ eV; 3 eV; 1 eV; -3 eV. The valence to conduction band transitions $d_{12}^k = d_{21}^k$ are implemented as in the main text (Eq. 6, Main Text). The valence band transitions are implemented with $d_{13}^k = d_{31}^k = 12$. The conduction band transitions are implemented with $d_{24}^k = d_{42}^k = 12$. All other dipole matrix elements are set to zero. This band structure reproduces the same OKE as the band structure used in Supplementary Fig. 3 and is very similar to the structure used in A. Schiffrin, *et al.*, Optical-field-induced current in dielectrics, Nature 2013 Vol. 493, 70 (2013).

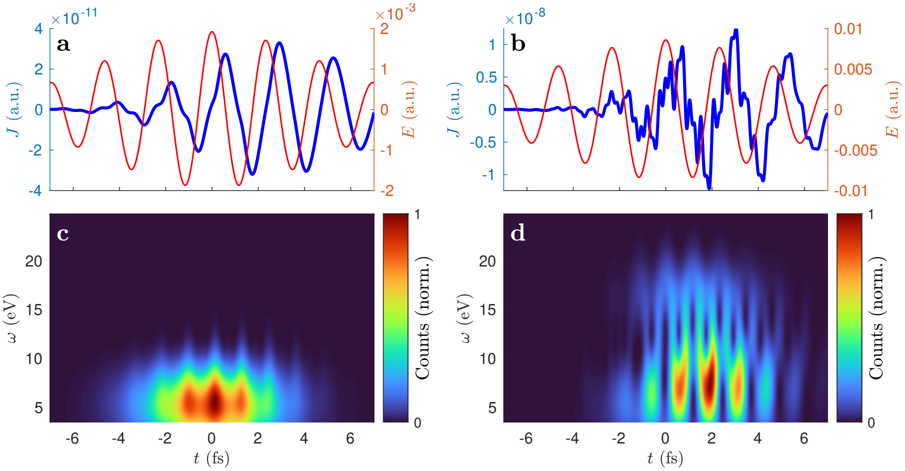

Supplementary Figure 5. The current in time-domain. Panels **a**, **b**, **c**, **d** are the same as in Fig. 6 (Main Text), but J instead of $P^{(\mathrm{NL})}$ is displayed.

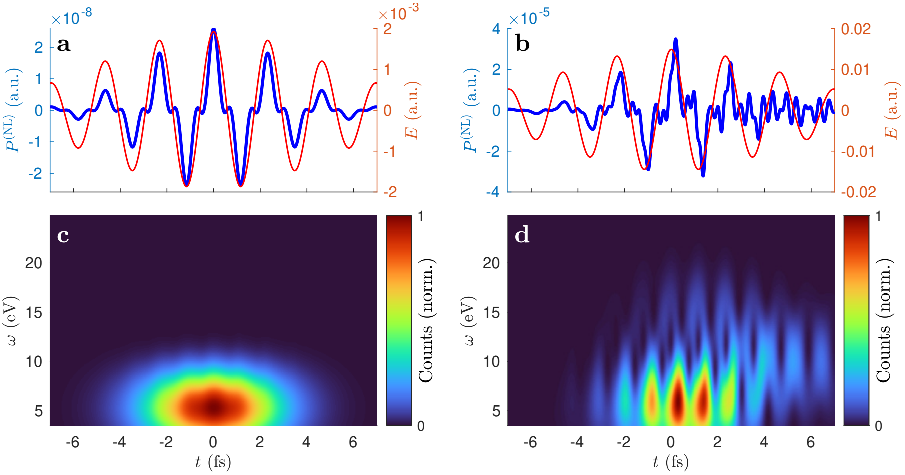

Supplementary Figure 6. The nonlinear polarization response without Bloch electron motion. Panels **a**, **b**, **c**, **d** are the same as in Fig. 6 (Main Text), but using the simplified calculation that neglects the motion of the Bloch electrons by omitting the coordinate transform $k \to k + \dot{A}$ in Eq.(1, Main Text). The peaks of $P^{(\mathrm{NL})}$ in **b** are not dented as compared to Fig. 6 (Main Text).
